# The legacy of ZikaPLAN: a transnational research consortium addressing Zika

**DOI:** 10.1080/16549716.2021.2008139

**Published:** 2022-04-04

**Authors:** Annelies Wilder-Smith, Elizabeth B. Brickley, Ricardo Arraes de Alencar Ximenes, Demócrito de Barros Miranda-Filho, Celina Maria Turchi Martelli, Tom Solomon, Bart C. Jacobs, Carlos A. Pardo, Lyda Osorio, Beatriz Parra, Suzannah Lant, Hugh J Willison, Sonja Leonhard, Lance Turtle, Maria Lúcia Brito Ferreira, Rafael Freitas de Oliveira Franca, Louis Lambrechts, Johan Neyts, Suzanne Kaptein, Rosanna Peeling, Deborah Boeras, James Logan, Helen Dolk, Ieda M Orioli, Andreas Neumayr, Trudie Lang, Bonny Baker, Eduardo Massad, Raman Preet

**Affiliations:** a Department of Epidemiology and Global Health, Umeå University, Umeå, Sweden; b Heidelberg Institute of Global Health, University of Heidelberg, Heidelberg, Germany; c London School of Hygiene & Tropical Medicine, London, United Kingdom; d Departamento de Medicina Tropical, Universidade Federal de Pernambuco, Recife, Brasil; e Departamento de Medicina Interna, Universidade de Pernambuco, Recife, Brasil; f Instituto Aggeu Magalhães, Fundação Oswaldo Cruz, Recife, Brasil; g NIHR Health Protection Research Unit for Emerging and Zoonotic Infections, Institute of Infection, Veterinary and Ecological Sciences University of Liverpool, Liverpool, UK; h Departments of Neurology and Immunology, Erasmus Universitair Medisch Centrum Rotterdam, The Netherlands; i Department of Neurology, Johns Hopkins School of Medicine, Baltimore, Maryland, USA; j Universidad del Valle, Cali, Colombia; k Institute of Infection, Immunity & Inflammation, University of Glasgow, Glasgow, UK; l Hospital da Restauração, Recife, Brazil; m Oswaldo Cruz Foundation, Recife, Brazil; n Insect-Virus Interactions Unit, Institut Pasteur, UMR2000, CNRS, 75015 Paris, France; o KU Leuven Department of Microbiology, Immunology and Transplantation, Rega Institute for Medical Research, Laboratory of Virology and Chemotherapy, Leuven, Belgium; p The Global Health Impact Group, USA; q Centre for Maternal, Fetal and Infant Research, Institute for Nursing and Health Research, Ulster University, Ulster, United Kingdom; r RELAMC and ECLAMC at Genetics Department, Federal University of Rio de Janeiro, Brazil; s Department of Medicine, Swiss Tropical and Public Health Institute, Basel, Switzerland; t The Global Health Network, Centre for Tropical Medicine and Global Health, University of Oxford, Oxford, UK; u School of Medicine, University of Sao Paulo and Fundacao Getulio Vargas, Sao Paulo, Brazil

**Keywords:** Zika, congenital Zika syndrome, birth defect, epidemic preparedness, research capacity building, European Commission, microcephaly, Guillain-Barré Syndrome, encephalitis, sustainability

## Abstract

Global health research partnerships with institutions from high-income countries and low- and middle-income countries are one of the European Commission’s flagship programmes. Here, we report on the ZikaPLAN research consortium funded by the European Commission with the primary goal of addressing the urgent knowledge gaps related to the Zika epidemic and the secondary goal of building up research capacity and establishing a Latin American-European research network for emerging vector-borne diseases. Five years of collaborative research effort have led to a better understanding of the full clinical spectrum of congenital Zika syndrome in children and the neurological complications of Zika virus infections in adults and helped explore the origins and trajectory of Zika virus transmission. Individual-level data from ZikaPLAN`s cohort studies were shared for joint analyses as part of the Zika Brazilian Cohorts Consortium, the European Commission-funded Zika Cohorts Vertical Transmission Study Group, and the World Health Organization-led Zika Virus Individual Participant Data Consortium. Furthermore, the legacy of ZikaPLAN includes new tools for birth defect surveillance and a Latin American birth defect surveillance network, an enhanced Guillain-Barre Syndrome research collaboration, a de-centralized evaluation platform for diagnostic assays, a global vector control hub, and the REDe network with freely available training resources to enhance global research capacity in vector-borne diseases.

## Background

Global health research partnerships are increasingly taking the form of consortia of institutions from high-income countries and low- and middle-income countries that undertake programs of research. These partnerships differ from collaborations that carry out single projects in their manifold variety of goals, activities, including the nature of their management [1]. Such consortia primarily aim to enhance research collaboration between and within countries and continents by facilitating access to patient cohorts for shared data analyses, levering upon the strength of multidisciplinary and international research approach, and building up collaborative networks between research institutions across the globe.

After a cluster of children born with abnormally small head circumferences was detected in northeast Brazil in late 2015, and a public health emergency declared in early 2016 [2], the European Commission awarded three research consortia with global health research partnerships to urgently address the knowledge gaps related to Zika virus infections, and form a research network with Latin America. ZikaPLAN stands for ‘Zika Preparedness Latin American Network’ and was one of the three consortia [3]. ZikaPLAN is a consortium with more than 100 researchers from 26 institutions from Belgium, Brazil, Colombia, Cuba, France, Senegal, Switzerland, The Netherlands, UK, and USA, coordinated by the University of Umeå in Sweden. It is interlinked with the other two EU funded consortia ZIKAlliance and ZIKAction through co-managed and cross-cutting three joint work packages related to communication, management, ethics, networking and cohort studies. A detailed description on the geographic distribution and work packages was published at the beginning of the project [3] with a further mid-term update [4].

The ZikaPLAN project started in October 2016 and came to an end in May 2021. In this article, we exemplify the key learnings, achievements and impact of ZikaPLAN with a focus on the clinical and public health work, drawing upon the networks and resources as well as more than one hundred ZikaPLAN publications to date (available at the project website https://zikaplan.tghn.org/publications/).

## A Addressing congenital Zika syndrome

The etiologic agent triggering the epidemic of microcephaly cases was initially an open question [5,6]. A series of epidemiological studies advanced scientific thought by connecting the microcephaly cases to Zika virus (ZIKV) infections in pregnancy [7–9], ruling out alternative hypotheses, such as larvicide [10] and providing the first clinical description of a new disease, congenital Zika Syndrome [11,12]. As part of the response to the epidemic, teams of investigators based in the Brazilian states of Pernambuco, Rio de Janeiro, and Goiás initiated, with support from the ZikaPLAN Consortium and the Brazilian government, a series of prospective cohort studies of pregnant persons with rash and children with Congenital Zika Syndrome (CZS) to elucidate the risks associated with maternal ZIKV infections.

### Pregnancy cohort & vertical transmission studies

To identify cases of acute maternal Zika virus (ZIKV) infections, the Microcephaly Epidemic Research Group (MERG) in Pernambuco State described the serological markers of ZIKV and dengue virus (DENV) among mothers and neonates [7,8,13] and tested participants in the MERG Pregnant Women Cohort. The former study showed a high frequency of ZIKV exposure among mothers of microcephalic neonates and distinct patterns of ZIKV and DENV immune responses, as discernible by the neutralization test [13]. In the latter, women were tested at up to three timepoints during and after pregnancy using a combination of molecular and serologic assays, and the results were integrated in an evidence-graded diagnostic algorithm [14]. Among the pregnancies with suspected or confirmed ZIKV infections, 20% of ZIKV-exposed offspring presented with at least one clinical feature compatible with CZS, with absolute risks for microcephaly of 3%, neuroimaging abnormalities of 7%, neurologic abnormalities of 5%, and ophthalmologic abnormalities of 7% [15]. Interestingly, vertical transmission of ZIKV, placental features, and neurodevelopmental outcomes can be discordant between co-twins [16].

ZikaPLAN found low sensitivity of both ultrasonography [17] and amniocentesis [18] for prenatal CZS screening and affirmed the importance of comprehensive clinical assessment of neonates with suspected and confirmed ZIKV exposure during pregnancy. Sixty-five per cent of evaluable infants born to PCR-positive mothers were found to have serologic or molecular evidence of vertical transmission when tested within the first three months of life, with decreasing vertical transmission rates over the three trimesters (78% in first, 64% in second, and 48% in third) [19]. A similar temporal pattern was apparent in the frequency of children born small for gestational age and/or with symptoms consistent with CZS, with the highest rates of adverse outcomes in children born to women reporting rash (i.e. a common sign of acute ZIKV infection) in the first trimester [20]. Relative to ZIKV-negative neonates, ZIKV-positive neonates have a 5-times higher risk of presenting with microcephaly [21]. Lower socioeconomic position (i.e. indicated by lower maternal education, lower household income, and higher household crowding) was associated with increased odds of a child being born with congenital microcephaly [22]. Families residing in areas with poor living conditions had a higher prevalence of microcephaly compared with populations with better living conditions [23].

### Observational studies of children with congenital Zika syndrome

The ZikaPLAN studies provided unique insights regarding the natural history of CZS. Of note, the MERG pediatric cohort is one of the largest single cohort studies of children with CZS in the world. The MERG pediatric cohort study published several case series reporting on microcephaly [24], prenatal and postnatal brain imaging abnormalities [25,26], oropharyngeal dysphagia [27], early epilepsy [28,29] and rare complications such as cryptorchidism [30,31], endocrine dysfunctions [32], and adenoid hypertrophy [33] over the first years of life. Longer term follow-up of the prenatally exposed infants also found markedly elevated frequencies of seizures, ophthalmologic abnormalities, and hearing deficits in infants born with microcephaly or born small for gestational age relative to exposed infants born without microcephaly and normal or large for gestational age [34]. High frequencies of structural abnormalities and neurodevelopmental delays [35], as well as adverse outcomes detected using the General Movement Assessment tool [36], were also identified in ZIKV-exposed children born without microcephaly. Observations from this cohort have also shed light on the impacts of CZS on early nutrition and growth for ZIKV-exposed infants, including decreasing weight-for-age Z-scores in the first 14 days of life for hospitalized neonates [37], low rates of exclusive breastfeeding, altered body composition with a lower fat-free mass [38], and worsening anthropometric profiles by 12–23 months of age [39].

As the children in our cohorts have reached preschool age and beyond, new research is shedding light on the neurodevelopment of children with prenatal ZIKV exposure. A cross-sectional study of 274 children who were evaluated using the Survey of Wellbeing of Young Children neurodevelopment and behavior-screening instrument reported an association between a child’s head circumference and their likelihood of being ‘at risk of developmental delay,’ with 99% ‘at risk’ in the severe microcephaly group, 65% in the moderate microcephaly group, 14% in the ZIKV-exposed without microcephaly group, and 22% in the unexposed, neurotypical control group [40]. Furthermore, a cross-sectional study of 94 children found that ZIKV-exposed children with normal findings upon brain imaging had reduced odds of severe developmental delay, as indicated by a Bayley-III score of 70 or less, relative to children with abnormal neuroimaging (odds ratio, 95% confidence interval: 0.80, 0.70 to 0.91) [41]. Using the Bayley-III assessments provided evidence of a high frequency of developmental delays, particularly in the language domain, among children with prenatal ZIKV exposure but who were born without apparent symptoms of CZS at birth [42] and potential effect modification on the association between prenatal ZIKV exposure and neurodevelopment by socioeconomic position [22].

### Social impacts of congenital Zika syndrome

Between May 2017 and January 2018, ZikaPLAN undertook a series of in-depth qualitative interviews and a cross-sectional study to understand the social and economic impact of CZS in Pernambuco and Rio de Janeiro, Brazil [43,44]. Interviews with mothers suggest that the birth of a child with CZS can be conceptualized as a biographical disruption, requiring a reconfiguration of mothers’ and caregivers’ support systems and subsequent identities [45]. Interviews with caregivers and healthcare providers also highlighted gender inequalities in caregiving and bottlenecks in accessing healthcare services for children with disabilities [46] and reinforced the importance of trust and co-created hope in the acceptance of therapeutic interventions for children with CZS [47]. The cross-sectional study indicated that more than half of mothers of children with CZS reported at least mild levels of depression (51%), anxiety (53%), and stress (60%), all of which occurred at higher crude frequencies than among caregivers of children without disabilities [48].

### Prevention of congenital Zika syndrome

In response to the CZS threat, vector control and bite-prevention strategies are among the limited options currently available [49]. Pregnant women individual protection by use of repellent or wearing repellent-treated materials is likely to be a challenge even in the presence of enhanced messaging and education. Focus group discussions with pregnant women, women of child-bearing age and their male partners in Colombia highlighted significant gaps in the knowledge of key aspects of Zika disease and bite-prevention, lack of awareness of disease risk and misconceptions about repellent safety. However, health workers were identified as trusted sources of bite-prevention education, suggesting the need to equip them to deliver clear risk-benefit information of approved repellents [50].

### Data synthesis & sharing with international partnerships and WHO

In addition to reporting on the findings from individual cohorts, our team of investigators has also been actively engaged in wider evidence synthesis and data sharing efforts, leading to two systematic reviews [51,52] and contributing to four critical commentaries [6,53–55] and three planned individual participant data meta-analyses. The first systematic review collates evidence from 34 studies of ZIKV co-infections, and suggests that, while ZIKV co-infections, especially with other arboviruses, are frequently reported, they do not appear to materially alter the clinical presentation or enhance the severity of uncomplicated acute ZIKV disease [51]. The second systematic review of 32 studies on postnatal ZIKV infections in children and adolescents highlights the scant evidence base in this age range and the need for population-based studies quantifying the associations of pediatric ZIKV infections, especially those occurring in infancy, with neurologic complications and later neurodevelopmental outcomes [52]. Individual-level data from our cohort studies has also been shared for joint analyses as part of the Zika Brazilian Cohorts Consortium [56], the European Commission-funded Zika Cohorts Vertical Transmission Study Group [57], and the World Health Organization-led Zika Virus Individual Participant Data Consortium [58,59]. Joint analyses are ongoing.

*In summary, ZIkaPLAN has yielded some of the most robust estimates to date of the absolute risks associated with congenital Zika virus infections and provided unique insights regarding the prognosis and health needs of children born with Congenital Zika Syndrome.*

## B Setting up birth defect surveillance in Latin America

The Zika outbreak created more awareness about the need for effective sharing of documentary, software, online and training resources related to birth defects surveillance and research. In December 2018, the International Committee for Congenital Anomaly Surveillance Tools (comprising members from Europe, Latin America, Africa, Asia and the USA) was launched. The Global Birth Defects (GBD) website (https://globalbirthdefects.tghn.org/) is fully established. It hosts an inventory of existing birth defect surveillance resources as well as information on the Global Birth Defects Description and Coding App. This website nests within The Global Health Network (TGHN) website. The inventory of the GBD website covers a wide range of resources related to birth defects surveillance/research, prevention and care, in eight sections.

The Global Birth Defects Description and Coding (GBDDC) App is a companion tool that can be used by a wide range of health professionals contributing to birth defect surveillance, research and training in low resource settings [60]. It is designed to assist with the description and coding of major externally visible birth defects during the neonatal period. Special modules relate to microcephaly and Congenital Zika Syndrome diagnosis. It is not intended to replace referral of the baby for clinical diagnosis or to inform care options requiring clinical input. The GBDDC App has been designed in two versions:

- the Basic Version designed for use by persons with an interest in understanding/improving birth defect diagnosis or coding, including for training purposes,
- the Surveillance Version which is an extension of the basic version that allows recording of anonymous data for each baby for use in surveillance/research systems.

The App has been translated into Spanish and Portuguese. The Basic Version of the App was launched in October 2019, is now available for download in the Google play store and Apple store, with adaptations possible to suit specific needs. The GBDDC App will be taken over by WHO to form part of the Birth Defects Surveillance toolkit. For reaching various stakeholders and communities, a short video ‘Innovation through Collaborations’ was produced where GBDDC is featured (https://www.youtube.com/watch?v=Mdf7F8i7azg&t=5s) (Table 1).

ZikaPLAN enabled the development of a Latin American network of congenital malformation registries (ReLAMC) [61]. Data sharing rules were established, and this led to the first publication of data on microcephaly and other congenital anomalies from all 12 participating countries for the period 2017–2019, laying the foundation of greater data harmonization in future. Eight RegLAMC registries collaborated in a study of microcephaly and congenital infections with data from the period 2010–2017 (submitted for publication). This showed increases in microcephaly prevalence from two-fold to 70-fold in countries and regions, which experienced the Zika epidemic, with some evidence of improved awareness and reporting of microcephaly continuing beyond the epidemic. The need to collect more data about preventable congenital infections other than Zika was also highlighted for action.

During the Zika pandemic, WHO/PAHO collected notifications of Zika infections and Congenital Zika Syndrome for all countries globally. A summary of these data was collated for the 47 countries in the Americas and the Caribbean, and the strengths and deficiencies of the reporting system were evaluated [62]. By 4 January 2018, 3617 confirmed CZS cases had been reported from 25 countries, 82% from Brazil and 7% from Colombia. There were high CZS rates (above 0.5 per 1000 births) in a number of Caribbean Island communities, with evidence of both severe under-reporting of CZS, and of Zika virus infections among pregnant women.

*In summary, ZikaPLAN has enhanced birth defect surveillance in Latin America and beyond by creating and developing networks and producing tools such as the Global Birth Defects Description and Coding App.*

## C Investigating neurological complications of Zika in adolescents and adults

One of the most prevalent systems seriously affected by ZIKV is the nervous system both through direct infection of foetal and adult neural cells by ZIKV and through immune-mediated damage of the nervous system. In co-operation with the clinical studies conducted within ZikaPLAN, viral isolates, human tissue and blood samples were also collected for laboratory-based investigations into mechanistic pathways underlying the neurological manifestations of ZIKV beyond congenital Zika syndrome. These included exposure of cell lines and neural tissues in the post-neurogenesis time window, *in vitro* and *in vivo*, to ZIKV isolates followed by examination of cell viability, function and downstream molecular cascades. Also, human samples collected from ZIKV cases and controls were examined in a range of assay systems for immunological factors that might drive ZIKV neurological disease.

Using myelinating cultures, we first observed that glial cells in the central nervous system (CNS) were much more susceptible to ZIKV infection compared with cells in the peripheral nervous system (PNS), where infection was minimal [63]. Within the CNS, myelin-forming oligodendrocytes were particularly vulnerable to infection, which induced cell death and myelin loss, accompanied by axonal degeneration in the absence of any observable injury to, or ZIKV infection of neuronal cell bodies. When early postnatal mice were infected with ZIKV *in vivo*, we again saw a propensity for oligodendrocyte infection, with relative sparing of neurons [64]. These studies collectively indicate that direct infection of peripheral nerve is unlikely to account for the development of Guillain-Barré syndrome (GBS) and that amongst the CNS manifestations of ZIKV infection clinicians should be particularly alert to de- and dys-myelinating syndromes and pathologies.

ZIKV replicates more efficiently in neural progenitor cells than in postmitotic neurons. ZIkaPLAN identified host factors such as BAF45b, which regulates differentiation of neural progenitor cells to post-mitotic neurons [65]. Given their function in cell and tissue differentiation, regulators such as the BAF complex may be important determinants of tropism and pathogenesis of arthropod-borne flaviviruses, which should be further investigated.

To explore the molecular mechanisms, we did genetic screens using CRISPR/Cas9 technology, mass spectrometry of pull-down assays and transcriptional analysis [66]. Genes and sets of genes identified included those involved in antiviral responses, neuroinflammation, demyelination and regulation of gene expression and differentiation. ZikaPLAN`s immunological studies on clinical samples attempted to identify anti-nerve autoimmune factors, notably autoantibodies that might drive the development of GBS. The major class of previously identified GBS autoantibodies to nerve glycolipids including gangliosides do not appear to be present in ZIKV-GBS sera, despite extensive screening of multiple GBS and control cohorts [67]. Further exploration of unknown autoantigen targets in ZIKV-GBS is being pursued through screening peripheral nerve cultures, cell lines and sectioned tissues for antibody-binding events. Furthermore, T cell responses have been characterised from acute phase lymphocytes in clinical samples, along with case characterisation through arboviral screening protocols. We found that T cell responses triggered by pre-existing dengue virus infection are protective against Zika virus infection [68]. In a mouse model, specific T cells to Japanese encephalitis virus, another flavivirus, could also protect against Zika [69].

### The spectrum of Zika virus-associated neurological disease

ZikaPLAN launched a specific case–control study to determine the association between GBS and ZIKV and other arbovirus infections such as chikungunya and dengue. The case–control study, named ‘IGOS-Zika’, was conducted in several hospitals across Brazil, Argentina and Malaysia. To enhance harmonization of data collection of GBS patients across different study groups, the IGOS protocol was also used as template in the observational cohort study on GBS in Colombia in the Neuroviruses Emerging in the Americas Study (NEAS) [70]. ZikaPLAN was the first to identify that Zika virus infection can result in a mixed picture of both central and peripheral nervous system disease [71,72], and we reported on several rare neurological complications [73–75]. We found that whilst chikungunya virus infection was more often associated with central nervous system disease, especially myelitis, Zika was more often linked to peripheral nervous system disease, especially Guillain-Barré syndrome (GBS). We recognized a high frequency of co-infection with Zika and chikungunya virus in patients in our studies with neurological disease, underscoring the need for clinicians to investigate for evidence of both pathogens. Our study of 71 GBS cases found that almost one-third of cases had laboratory evidence of a recent infection with ZIKV or chikungunya virus [67]. Most patients with a recent arbovirus infection had a sensorimotor, demyelinating GBS. We did not find a specific anti-glycolipid antibody signature in association with arbovirus-related GBS, further elaborated upon in our systematic review [76]. During the Zika epidemic, it became apparent that no international guideline existed for the management of GBS. To aid clinicians in managing GBS patients, ZikaPLAN developed the first consensus-based international clinical guideline for the diagnosis and management of GBS [77].

*In summary, ZikaPLAN contributed to the understanding of the pathomechanism of Zika virus related neurological complications and developed a 10-step guidance for the clinical management of Guillain-Barre Syndrome.*

## D Exploring factors that led to the emergence of congenital Zika syndrome as a public health problem

The global emergence of ZIKV revealed the unprecedented ability for a mosquito-borne virus to cause congenital birth defects such as microcephaly. Sporadic human Zika virus (ZIKV) infections have been recorded in Africa and Asia since 1950s. Major epidemics occurred only after ZIKV emerged in the Pacific Islands and spread to the Americas [78]. A puzzling aspect of ZIKV emergence is that all human outbreaks and birth defects to date have been exclusively associated with the Asian ZIKV lineage, despite a growing body of laboratory evidence pointing towards higher transmissibility and pathogenicity of the African ZIKV lineage. Whether this apparent paradox reflects the use of relatively old African ZIKV strains in most laboratory studies was unclear. ZikaPLAN researchers assembled a panel of low-passage ZIKV strains representing the current global viral diversity to experimentally evaluate their fitness differences. ZikaPLAN found that recent African ZIKV strains largely outperformed their Asian counterparts in mosquito transmission kinetics experiments, which translated into a markedly higher epidemic potential in outbreak computer simulations [79]. In addition, African ZIKV strains were significantly more lethal than Asian ZIKV strains in immunocompromised adult mice. Finally, prenatal infection of immunocompetent mouse embryos with an African ZIKV strain resulted in embryonic death, whereas it caused microcephaly with Asian ZIKV strains. Together, these results demonstrate the high epidemic potential and pathogenicity of recent ZIKV strains from Africa. Importantly, they also imply that the African ZIKV lineage could more easily go unnoticed by public health surveillance systems than the Asian ZIKV lineage due to its propensity to cause foetal loss rather than birth defects [80].

The high epidemic potential of African ZIKV strains is counter-intuitive because continental Africa has remained free of large-scale Zika outbreaks so far. We hypothesized that this discrepancy could reflect genetic differences in the vector populations between Africa and the rest of the world. The mosquito *Aedes aegypti* is a species native to Africa that invaded most of the world’s tropical belt over the past four centuries. This geographical range expansion followed the evolution of a ‘domestic’ form of *Ae. aegypti* that specialized in biting humans and breeding in human water-storage containers. We experimentally challenged several field-derived *Ae. aegypti* colonies from across the species range with our worldwide panel of ZIKV strains [81]. *Ae. aegypti* populations from their ancestral range in sub-Saharan Africa were significantly less susceptible to ZIKV infection, regardless of the virus strain, than the globally invasive populations from tropical America and Asia. Among the African populations, ZIKV susceptibility was positively correlated with their proportion of ‘domestic’ genetic ancestry. Genetic analysis of a cross between African and non-African mosquitoes further identified genomic regions associated with differences in ZIKV susceptibility. Finally, mouse studies showed that African mosquitoes were substantially less likely to transmit ZIKV from a viremic host than their non-African counterparts. These results indicate that the recent evolution and range expansion of *Ae. aegypti* outside Africa was accompanied with a marked increase in its ability to carry ZIKV. This finding may contribute to explaining why ZIKV has disproportionately caused outbreaks outside Africa.

Introduction and dispersal of ZIKV on the Pacific Islands were preceded by an extended period of relatively silent transmission in South-East Asia, enabling the virus to expand geographically and evolve adaptively before its unanticipated introduction to immunologically naive populations on the Pacific Islands and in the Americas [82]. Specific biological determinants of the explosive epidemic nature of ZIKV have not been identified. The genetic regions of ZIKV which are most homologous to the *Aedes* spp. borne dengue viruses while three large areas within the nonstructural protein encoding regions contain sequences more similar to *Culex*-borne antigenic complex III flaviviruses such as West Nile, Japanese encephalitis and Australasian flaviviruses [78]. ZikaPLAN researchers hypothesized that this incongruence reflects interspecies recombination resulting in ZIKV evasion of cross-protective T-cell immunity. Our findings demonstrate that the ZIKV proteome is a hybrid of Aedes-borne dengue virus (DENV) proteins interspersed amongst Culex-borne flavivirus proteins derived through independent interspecies recombination events. We propose that by acquiring cytotoxic T-cell epitope-rich regions from Culex-borne flaviviruses, ZIKV evaded DENV-generated T-cell immune cross-protection. Thus, Culex-borne flaviviruses, including West Nile virus and Japanese encephalitis virus, might induce cross-protective T-cell responses against ZIKV. This would explain why explosive ZIKV epidemics occurred in DENV-endemic regions of Micronesia, Polynesia and the Americas where Culex-borne flavivirus outbreaks are infrequent and why ZIKV did not cause major epidemics in Asia where Culex-borne flaviviruses are widespread [78].

*In summary, ZikaPLAN elucidated the factors that led to the emergence of congenital Zika syndrome in Latin America and notAfrica*.

## E ZikaPLAN’s research on Zika’s geographic spread via international travel and its epidemic trajectory

Travellers contributed substantially to the rapid spread of ZIKV [83]. ZikaPLAN collaborated with GeoSentinel, a global surveillance network of returning travellers [84,85]. A literature review of the GeoSentinel publications related to Zika showed that Zika was first reported by GeoSentinel already in 2012, and notifications peaked in the years 2016–17 reflecting the public health emergency in the Americas at the time [86]. GeoSentinel sites reported 525 ZIKV cases from 2012 through 2019 with a clear decline in travellers after the 2016 outbreak [87]. Main countries of exposure were the Caribbean Islands and Latin America. However, Zika cases have also been described outside of the Americas [88–91] and sporadically also still occur in Latin America [92]; hence, pre-travel advice and guidance need to continuein particular, for pregnant travellers [49,93]. ZikaPLAN has developed a smartphone App-based sentinel surveillance system for (i) the assessment of the incidence of arboviral-related symptoms in travellers and (ii) the risk assessment/modelling of travel-related introduction of arboviruses to vector-infested regions of Europe. ZikaPLAN also developed `baited filter paper-based mosquito traps´ to assess the arbovirome (i.e. to identify the spectrum of arboviruses in local mosquito populations in tropical and subtropical countries for sentinel surveillance.

Applying a spatio-temporal dynamic transmission model for ZIKV infection to determine key transmission parameters and projected incidence in 90 major cities within 35 countries, ZikaPLAN researchers together with ZIKAlliance modellers predicted that the ZIKV epidemic would be by and large over within Latin America within a matter of a year [94]. Local low levels of transmission are probable, but the estimated rate of infection suggests that most cities have a population with high levels of herd immunity. However, in 10 years as population herd immunity wanes, it is very plausible that Zika will return.

*In summary, ZikaPLAN studied mobility networks and traveller’s sentinel surveillance to anticipate the projected trajectory and global spread of Zika.*

## F ZikaPLAN’s legacy with regards to enhancing research capacity and collaborative networks between Europe and Latin America

ZikaPLAN’s final objective, as outlined by the European Commission in its call, was for the Zika outbreak research response effort to grow into a sustainable Latin-American network for emerging infectious diseases research preparedness. True transparent research partnerships are needed to ensure successful research and sharing of data. Such partnerships require trust, mutual benefit and sharing and equitable standing of all partners. Trust can only be built up by relationships. Relationships were fostered by EU-funded ZikaPLAN meetings, which took place every year in different ZikaPLAN sites in Brazil, Cuba, Colombia, and the UK. We engaged in capacity building in laboratory and clinical research, collaborated with existing networks to share knowledge, and set up new networks and training resources for capacity building. In the following, we describe some selected platforms, networks and web-based hubs that ZikaPLAN .established

### ZikaPLAN’s diagnostic evaluation platform and biobanking network

Biobanking and diagnostic evaluation networks play a critical role in providing the necessary resources and mechanism to facilitate and accelerate diagnostic development and evaluation so that quality-assured tests can be deployed without delay by countries for future pathogens of epidemic potential [95]. ZikaPLAN’s goal was to accelerate the development and evaluation of Zika diagnostics for clinical and surveillance use through the establishment of a biobanking network to guarantee access to well-characterized clinical specimens and capacity for independent evaluations [3,4]. Six sites in Africa, Asia and Latin America with well-characterized clinical specimens were selected to evaluate Zika diagnostic tests using a standardised protocol (**Figure 1**). All sites are proficient in performing reference standard tests and compliant with Good Clinical Practice/Good Clinical Laboratory Practice. ZikaPLAN partnered with UNICEF and USAID to evaluate various Zika and multiplex arbovirus diagnostic tests. Development and evaluation of diagnostics for diseases of epidemic potential are often funded during an epidemic and left unfinished once the epidemic is over when funding is no longer available. UNICEF and USAID made an advance purchase commitment with ZikaPLAN to ensure the development and availability of quality-assured diagnostics for diseases of epidemic potential. ZikaPLAN diagnostic evaluation platform has already served as a model platform for the COVID-19 pandemic [96]. The biobanking network and its governance structure were leveraged upon to evaluate SARS-CoV-2 assays [95], dengue assays and rapid diagnostic tests [97–100].

### Creation of Latin American network for congenital malformation surveillance (ReLAMC)

To increase the epidemiologic surveillance in Latin America, a Latin American network of congenital malformation registries (ReLAMC) was created [61] building on the foundation of another surveillance network, ECLAMC (Estudio Colaborativo Latino Americano de Malformaciones Congénitas/Latin American Collaborative Study of Congenital Malformations). ECLAMC is an active hospital network of birth defects registries throughout South America. ReLAMC integrates ECLAMC but expands the network of registries to include not only hospitals but also national and regional registry systems, thus enhancing the research potential for the entire continent. The ReLAMC database was based on that used by the International Clearinghouse for Birth Defects Surveillance and Research and the European Surveillance of Congenital Anomalies (EUROCAT) model for the initial design of the Terms of Agreement, data sharing options, the use of data quality and public health indicators, and web page contents, particularly prevalence. Twelve countries in the Americas are participating, including five national registries, six regional registries, and various hospitals.

### International networks for investigating Guillain-Barré syndrome (GBS) and other neurological complications of arboviral diseases

ZikaPLAN built upon the International GBS Outcome Study (IGOS), the largest ongoing international observational cohort study on GBS, coordinated by Erasmus University in Amsterdam in the Netherlands [101] (https://gbsstudies.erasmusmc.nl/). Together with REDe, funding was acquired to create a dedicated GBS Knowledge Hub on The Global Health Network (https://rede.tghn.org/gbs-flowchart-sample/). Furthermore, when COVID-19 emerged as the next public health emergency of international concern, the IGOS network was leveraged upon to investigate the relationship between GBS and COVID-19, showing the ability of the IGOS network to quickly respond prepare for future outbreaks of infectious disease related to GBS [102]. The neurological networks set up through ZikaPLAN were critical in the successful application to the UK National Institute for Health Research (NIHR) for £2.3 M to establish ‘Brain Infections Global’ (an NIHR Global Health Research group on acute brain infections) and the subsequent Global Covid Neuro Network, established at the start of the COVID-19 pandemic. This network has applied approaches developed through ZikaPLAN to studying neurological COVID-19 disease, using standardised case definitions to harmonise data reporting [103].

### Global vector hub

An early version of The Global Vector Hub (GVH) was launched in June 2020, with a full version to follow in 2021 (https://globalvectorhub.lshtm.ac.uk/). This open-access online platform has a repository for entomological data, resources section, and networking features intended to establish a community of practice for vector control researchers and workers. The site aims to strengthen capacity building and data sharing for vector control globally. It currently has registered users from 74 countries and datasets have a current focus on *Aedes* in South and Central America, which will expand to other geographies and mosquito species soon. The GVH has directory of more than 100 training courses in medical entomology from all WHO regions, in collaboration with WHO Special Programme for Research and Training in Tropical Diseases. GVH co-created a Massive Open Online Course (MOOC) entitled ‘The Global Challenge of Vector Borne Diseases and How to Control Them’, which was launched in September 2020. GVH published a topical opinion article that brought together a broad coalition of global vector experts to discuss the impact of the COVID-19 pandemic on dengue control efforts [104]. To ensure accessibility and visibility, the GVH website is hosted by the Global Health Network.

### REDe

REDe is a ‘community of practice’ hosted by the Global Health Network (TGHN) with the purpose to accelerate and streamline research, through a digital platform. This online science park (https://rede.tghn.org/) is where research groups can benefit from collaborative working, knowledge sharing and dissemination. It also provides a platform for capacity development and resources. The flagship area for capacity development is the training centre, with 250,000 modules taken, embedded within the Global Health Trials section of the platform. A resource of particular interest for research methodology is the ‘Process Map’ toolkit, which currently provides systematic guidance for planning global health research projects and is being repurposed for use in tracking new studies. REDe also fosters collaboration within ZikaPLAN, which led to three knowledge hubs launched within TGHN (where REDe is hosted); Brain Infections Global, Global Birth Defects and The Global Vector Hub (Table 1). Online training has been successful with over 265,000 research skills courses taken regionally and 17 new courses have been developed with the University of Liverpool on identifying and diagnosing a range of neurological infectious diseases. Many of these resources and courses are available in Spanish and Portuguese to maximise utility and accessibility. To ensure further capacity building beyond the ZikaPLAN funding period, a memorandum of understanding was signed with Fiocruz, Brazil, to continue making training courses and resources freely available.

### Dissemination

In collaboration with all our consortium members and new networks, three different on-line dissemination events were organized towards the end of the ZikaPLAN project (https://zikaplan.tghn.org/zikaplan-tools/webinars/). In addition, four thematic videos (Table 2) were released in April 2021, summarizing the legacy of ZikaPLAN with its aim to enhance a sustainable Latin American research preparedness network.

*In summary, ZikaPLAN has created a network of networks for capacity building across various disciplines and to ensure sustainability.*

## Outlook

While the Zika virus pandemic has ended, the devastating health and social impacts on children with CZS and their families continue. The world cannot forget these affected families and communities, even now when the COVID-19 pandemic is drawing all our attention. We need to continue to advocate for effective support services for children with CZS and their families. As ZikaPLAN’s 5-year funding period has come to an end, renewed funding will be needed to continue monitoring the unique infant cohort that ZikaPLAN, ZIKAlliance and ZIKAction successfully set up through EU funding. There is a public health imperative to follow up on the neurodevelopment milestones and get a better grasp of the long-term physical and socioeconomic consequences of Zika- affected children when these children turn 5 and older. Furthermore, the world needs to be prepared for a likely return of Zika [105]. ZikaPLAN has developed animal models for future research on Zika-specific therapeutics and Zika vaccine candidates [106] [107–109], Zika vaccine development must continue even in the absence of major Zika virus circulation so that the world has tools at hand should Zika re-emerge [110,111].

## Dedication

This manuscript is dedicated to Professor Peter Byass who sadly passed away on 17 August 2020. He was instrumental in setting up the first EU-funded dengue research consortium hosted by the Umeå Centre of Global Health Research, Umeå University, Sweden. Through the established networks of DengueTools, we were able to rapidly re-create and broaden a related consortium – the Zika research consortium ZikaPLAN – as soon as Zika was declared a public health emergency of international concern. In fact, much of the proposal writing for ZikaPLAN took place in Peter’s home in mid-April 2016. We are thankful for the guidance received by Professor Byass during the proposal writing, and the warm hospitality by Mrs Byass during the stay.

## Paper context

Global health research partnerships with institutions from high-income countries and low- and middle-income countries are one of the European Commission’s flagship programmes. Here, we report on the ZikaPLAN research consortium funded by the European Commission by highlighting main achievements and outputs that will lead to the legacy of ZikaPLAN.
